# Supplementary material for: Identification of photoperiod-induced specific miRNAs in the adrenal glands of Sunite sheep (Ovis aries)
Source: Front Vet Sci. 2022 Jul 22;9:888207. doi: 10.3389/fvets.2022.888207 (PMC9354845; doi:10.3389/fvets.2022.888207)
Supplement: Supplementary file 1 [file Data_Sheet_1.ZIP › Supplementary file/miRDeep2 file.zip!/miRDeep2 file/Know_Structure/SPLP42A3/oar-mir-431.pdf]

miRBase precursor : oar-mir-431
Total read count : 690
oar-miR-431 read count : 98
remaining reads : 592

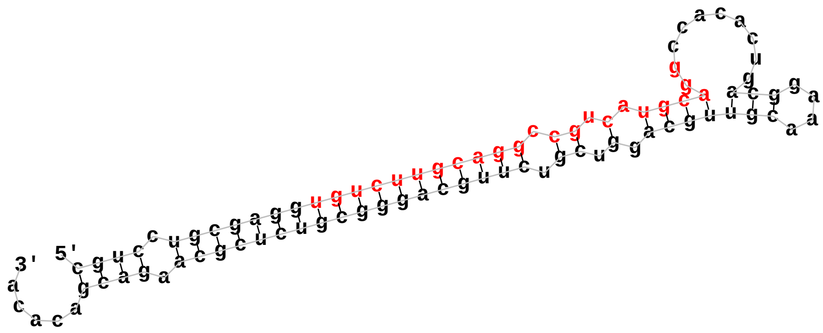

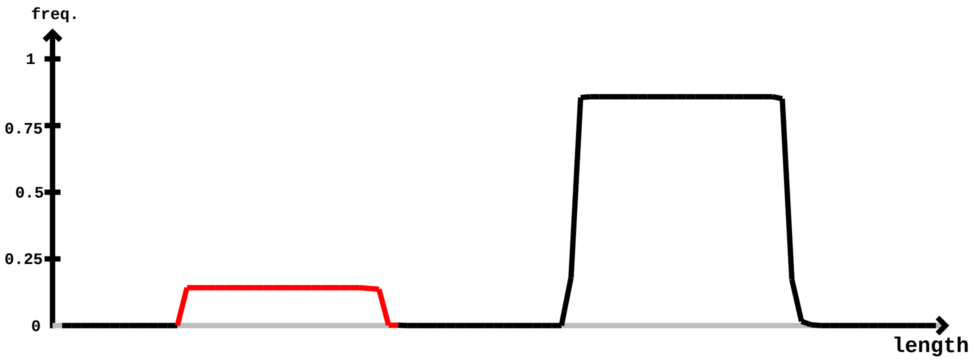


```
                oar-miR-431
5'- cguccugcgaggugucuugcaggccgucaugcaggccacacugacggaaacguugcaggucgucuugcagggcgucucgcaagacgacaca   -3'    exp
    ((((.((((((((((((((((((.((.(.((((..........(((....))))))).).)).))))))))))))))))))).)))).....    reads      mm         sample
    ............ugucuugcaggccgucaug............................................................    2          0          seq
    ............ugucuugcaggccgucaugc...........................................................    2          0          seq
    ............ugucuugcaggccgucaugca..........................................................    93         0          seq
    ............ugucuugcaggccgucaugcagg........................................................    1          0          seq
    ....................................................uugcaggucgucuugcagggcgu................    107        0          seq
    ....................................................uugcaggucgucuugcagggcguc...............    16         0          seq
    ....................................................uugcaggucgucuugcagggcgucu..............    1          0          seq
    .....................................................ugcaggucgucuugcagggcg.................    5          0          seq
    .....................................................ugcaggucgucuugcagggcgu................    361        0          seq
    .....................................................ugcaggucgucuugcagggcguc...............    90         0          seq
    .....................................................ugcaggucgucuugcagggcgucu..............    8          0          seq
    .....................................................ugcaggucgucuugcagggcgucuc.............    2          0          seq
    ......................................................gcaggucgucuugcagggcguc...............    2          0          seq
```
